# Low-Carbohydrate (Ketogenic) Diet in Children with Obesity: Part 2—Hormonal Effects of the Ketogenic Diet

**DOI:** 10.3390/children13030406

**Published:** 2026-03-14

**Authors:** Ivanka N. Paskaleva, Nartsis N. Kaleva, Teodora D. Dimcheva, Ivan S. Ivanov

**Affiliations:** 1 Department of Pediatrics “Prof. Dr. Ivan Andreev”, Faculty of Medicine, Medical University of Plovdiv, 4002 Plovdiv, Bulgaria; nartsis.kaleva@mu-plovdiv.bg (N.N.K.); ivan.ivanov@mu-plovdiv.bg (I.S.I.); 2 Department of Medical Informatics, Biostatistics and E-Learning, Faculty of Public Health, Medical University of Plovdiv, 4002 Plovdiv, Bulgaria; teodora.dimcheva@mu-plovdiv.bg

**Keywords:** ketogenic diet, obesity, insulin, thyroid hormones, cortisol, adiponectin, PCOS, children

## Abstract

**Highlights:**

**What are the main findings?**

• A relatively short-term “Well-formulated ketogenic diet” in children with obesity is associated with hormonal changes that promote weight loss and improve insulin sensitivity.
• The ketogenic diet leads to improvement of hormonal disturbances and restoration of the natural menstrual cycle in girls with PCOS.
• The ketogenic diet does not adversely affect thyroid function in children without pre-existing thyroid disease; however, in patients with autoimmune Hashimoto’s thyroiditis, changes in thyroid hormone levels may occur, requiring adjustment of replacement therapy.

**What are the implications of the main findings?**

• The ketogenic diet may be offered as an effective dietary intervention for motivated children with obesity and accompanying metabolic disorders.
• The ketogenic diet may be included as part of a comprehensive therapeutic approach in the treatment of girls with PCOS.
• During ketogenic nutrition, monitoring of TSH and thyroid hormone levels is recommended, particularly in patients with known pre-existing thyroid disease.

**Abstract:**

**Background:** For decades, the ketogenic diet has been successfully used for the treatment of obesity, metabolic syndrome, and type 2 diabetes. The mechanisms through which it affects metabolism are not fully understood, but the hormonal changes that occur during ketogenic nutrition are likely to play an important role. **Objectives:** To investigate the effect of the ketogenic diet on various hormones associated with obesity and the accompanying metabolic disorders in childhood. **Methods:** One hundred children aged 8–18 years with obesity were enrolled. After baseline anthropometric, biochemical, and hormonal testing, they followed a 4-month “well-formulated ketogenic diet.” Fifty-eight of them successfully completed the study with follow-up assessments. Among them, 8 girls had polycystic ovary syndrome (PCOS) and 7 children had Hashimoto’s autoimmune thyroiditis. **Results:** At the end of the 4-month period, there was a significant decrease in basal insulinemia (*p* < 0.0001) and in mean morning cortisol levels (*p* = 0.04), as well as an increase in adiponectin levels (*p* = 0.04). All girls with PCOS experienced spontaneous menstrual cycles, accompanied by a reduction in testosterone levels. TSH levels showed no change for the whole group (*p* = 0.13), but there was a significant decrease in T3 (*p* < 0.0001) and a mild increase in T4 (*p* = 0.05). Among patients with Hashimoto’s thyroiditis, TSH levels were significantly higher at the end of the study. **Conclusions:** A short-term, well-formulated ketogenic diet in children with obesity is associated with hormonal changes that support weight loss and improve insulin sensitivity. The diet shows particularly beneficial effects in girls with PCOS and may be considered as part of a comprehensive therapeutic approach in these patients. Monitoring thyroid function during ketogenic nutrition is advisable in patients with hypothyroidism and thyroid disorders.

## 1. Introduction

The Ketogenic Diet (KD) is a low-carbohydrate diet that includes a normal amount of protein to meet physiological needs, with fats and the ketones synthesized during their metabolism serving as the main source of energy [1]. This leads to a series of metabolic, biochemical, and hormonal changes in the body that resemble those occurring during prolonged fasting. These changes affect a number of pathological processes and diseases, which is why the KD is increasingly used in clinical practice. Some researchers refer to it as a metabolism-based therapy [1].

The KD was introduced into clinical practice for the treatment of epilepsy more than 100 years ago by Dr. Wilder at the Mayo Clinic [2]. In recent decades, various forms of this diet have been adopted in an increasing number of medical fields. There is a growing number of publications reporting successful treatment not only of a wide range of neurological diseases, but also positive outcomes in patients with endocrine, oncological, psychiatric, and other conditions and disorders [3,4,5,6].

The pathophysiological mechanisms through which KD affects all these different diseases are not fully understood. It is known that KD influences brain metabolism and neurotransmitters [7,8,9], improves cellular and mitochondrial biogenesis [10], reduces oxidative stress, and induces anti-inflammatory, neuroprotective, and epigenetic effects [11,12]. More recent studies highlight the significance of its effect on the gut microbiome [13].

Undoubtedly, the hormonal effects of KD are among the important mechanisms underlying its impact, especially in endocrine diseases and disorders such as obesity, metabolic syndrome, and type 2 diabetes [14,15,16]. Studies of KD in patients with obesity typically demonstrate a reduction in insulin levels [17,18,19]. This is considered an important mechanism explaining the beneficial effects of the diet in diseases associated with insulin resistance. There are relatively fewer studies examining the effects of KD on thyroid hormones, cortisol, and others. Some studies in children with epilepsy who follow a strict long-term KD have found that hypothyroidism may develop in some cases and recommend monitoring thyroid hormones in such patients [20,21]. Reports of beneficial effects of KD in polycystic ovary syndrome (PCOS) are also intriguing—this is a hormonal disorder that affects adolescent girls and is associated with fertility issues, psycho-emotional problems, increased risk of obesity, diabetes, and reduced quality of life [22,23,24].

One of the goals of our study on the effects of KD in children with obesity was to evaluate the diet’s influence on various hormonal levels in patients, as this could contribute to a more detailed understanding of the complex mechanisms through which this dietary approach affects a wide range of health disorders.

## 2. Materials and Methods

The materials and methods used in the study were described in detail in our first article on the effect of the diet on the children’s anthropometric measurements and indicators of insulin resistance, metabolic syndrome, and impaired metabolic health [25].

We present them briefly here as well.

### 2.1. Participant Selection and Study Design

One hundred children aged 8 to 18 years were selected to participate in the study. They were admitted for investigations at the Department of Pediatrics of University Hospital “St. George”, Plovdiv, between 2021 and 2023. Inclusion criteria were the presence of obesity (according to WHO criteria—BMI ≥ 2SD from the mean for gender and age) and at least one criterion for impaired metabolic health: abdominal obesity, impaired fasting blood glucose, primary arterial hypertension, dyslipidemia, hyperinsulinemia, hyperuricemia, hepatic steatosis, or polycystic ovarian syndrome. Exclusion criteria were proven adrenal gland dysfunction, pituitary pathology, congenital metabolic disease, treatment with medication causing insulin resistance, or contraindication for KD: familial (genetic) hypercholesterolemia, nephro- or cholelithiasis, history of pancreatitis.

**Figure 1:**
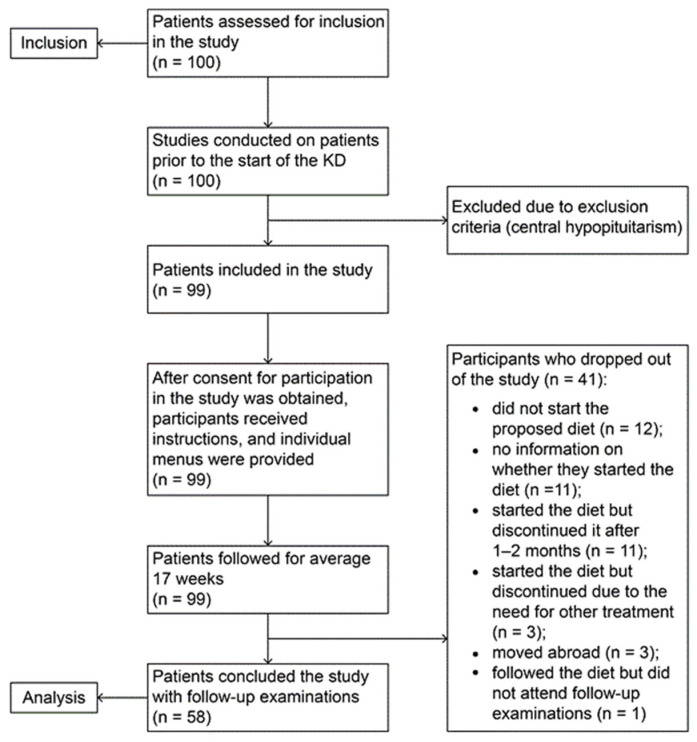
Flowchart of the study protocol.

Ninety-nine children were included in the study (1 patient was excluded due to exclusion criteria). After signing informed consent, patients underwent initial anthropometric, clinical, laboratory, and ultrasound examinations. All received detailed instructions on how to follow the diet. The patients were monitored over a period of 4 months, with families submitting weekly dietary reports electronically. Compliance with the diet was additionally monitored by measuring the level of βHB at home using the provided Care Sens Dual biosensor systems. During the follow-up period, 41 patients dropped out of the study. A total of 58 children who successfully completed the study after 4 months underwent second clinical, laboratory, and ultrasound examinations to assess the effect of dietary intervention (Figure 1).

The only recommended medications during the diet treatment were antihypertensive drugs for children with severe arterial hypertension at the pediatric cardiologist’s discretion, and L-thyroxine for patients with hypothyroidism. None of the patients took insulin sensitizers during the intervention. Girls diagnosed with polycystic ovarian syndrome did not receive hormonal therapy.

### 2.2. Characteristics of the Group of 58 Patients Who Completed the Study (Table 1)

In the interpretation of the results, it was taken into consideration that the 58 patients who completed the study represented a heterogeneous cohort, including both sexes, various stages of pubertal development, and differing levels of adherence to the prescribed dietary regimen—factors that were assumed to potentially affect the study outcomes. Therefore, patients were stratified according to sex, age, and dietary compliance, and all evaluated parameters were subsequently reanalyzed within these sub-groups.

The 58 patients who completed the study with follow-up assessments ranged in age from 8 to 18 years (mean 13.79 ± 0.34) at the start of the diet, including 35 (60.34%) boys and 23 (39.66%) girls. They were distributed into three age groups: 8 (13.79%) children in the age range 8–10 years, 25 (43.10%) children aged 11–15 years, and 25 (43.10%) aged 16–18 years. According to the compliance with the diet, the patients were divided into 3 groups: patients with good, moderate and poor compliance. Our assessment was based on information provided by parents, reported menus in food diaries, and measured values of beta-hydroxybutyrate (βHB). Patients with good compliance (strictly adhering to the diet) were 26 (44.83%), those with moderate compliance (following the diet with some deviations and exceeding the recommended carbohydrate intake) were 15 (25.86%), and those with poor compliance (frequent violations of the diet and multiple consumptions of inappropriate foods) were 17 (29.31%).

**Table 1:** Characteristics of the study group before the KD.

Variable	N = 58
Age, years (mean ± SD)	(13.79 ± 2.63)
Age groups, n (%)	
8–10 years	8 (13.79)
11–15 years	25 (43.10)
16–18 years	25 (43.10)
Gender, n (%)	
Female	23 (39.66)
Male	35 (60.34)
Anthropometric characteristics	
Mean weight/kg (SD)	89.43 (±3.30)
Mean BMI/kg/m^2^ (SD)	33.35 (±7.29)
Mean waist-height ratio (SD)	0.64 (±0.09)
Comorbidities, n (%)	
Metabolic syndrome	33 (56.89%)
One or more criteria for impaired metabolic health	25 (43.10%)
Polycystic ovary syndrome	8 (34.78%)
Primary arterial hypertension	27 (46.55%)
Hepatic steatosis	39 (67.24%)
Hashimoto’s autoimmune thyroiditis	7 (12.07%)
Compliance with the diet, n (%)	
Good	26 (44.82)
Moderate	15 (25.86)
Poor	17 (29.31)

### 2.3. Ketogenic Diet

The proposed diet is a “Well-formulated ketogenic diet”, in accordance with the guidelines of S. Phinney and J. Volek [26], with the following composition, structure, and recommendations:

- Carbohydrate intake: up to 40 g per day, divided into 10–13 g per meal.
- Protein intake: 1–1.5 g per kilogram of ideal body weight per day.
- Fat intake: enough to induce satiety without excessive consumption.
- Three to four meals per day: breakfast, lunch, dinner, and a small afternoon snack if hungry.
- Calorie counting is not necessary. Patients should eat until satisfied while adhering to the guidelines given in the individual menu. Skipping a meal is permissible if not hungry, but prolonged and intentional starvation is not recommended.
- Preference for natural foods: meat, fish, full-fat dairy products, eggs, low-carbohydrate vegetables, and a small number of low-carb fruits.
- Avoidance of processed, packaged foods, soft drinks, sweetened juices, and liquids.
- Fluid intake without sugar: 30–40 mL per kilogram per day.

### 2.4. Clinical Investigations

The focus of the clinical examination was on the following:

- Skin and its appendages (acanthosis nigricans, striae, acne, and the presence of increased hair in androgen-dependent areas in pubertal girls).
- Distribution of increased adipose tissue in different parts of the body.
- Cardiovascular system: measurement of heart rate and rhythm by auscultation; measurement of blood pressure with an age-appropriate sphygmomanometer under standard conditions.

### 2.5. Laboratory Investigations

- Oral glucose tolerance test with measurement of blood glucose and insulin at 0, 30, 60, and 120 min.
- Complete blood count, glycated hemoglobin, analyzed on an automated hemato-logical analyzer Advita 2120, Siemens Healthcare Diagnostics INC., Erlangen, Germany.
- Biochemical parameters: Lipid profile (total cholesterol, LDL cholesterol, HDL cholesterol, triglycerides), transaminases (ALT, AST), gamma-glutamyl transferase (GGT), urea, creatinine, uric acid, analyzed using original turbidimetric and immunoturbidimetric programs on a biochemical analyzer AU 480, Olympus; Beckman Coulter, Inc., Co Clare, Ireland.
- Hormones: Insulin, thyroid hormones (TSH, T3, T4), cortisol, testosterone, LH, FSH, analyzed using chemiluminescent immunoassay (CLIA) on an automated immunochemical analyzer Access 2, Beckman Coulter, Inc., Ireland.
- Adiponectin level was measured using the BioVendor Human Adiponectin ELISA test (BioVendor, Asheville, NC, USA).
- In 17 girls with advanced pubertal development, prior to starting the diet, the LH/FSH ratio and testosterone levels were measured (on days 3–5 of the menstrual cycle), and a detailed history was taken regarding family predisposition and menstrual cycle characteristics in order to identify those with polycystic ovary syndrome (PCOS).
- Serum levels of beta-hydroxybutyrate were measured once a week using the CareSens Dual Blood Glucose and Ketone Monitoring System.

### 2.6. Statistical Analysis

Continuous variables are expressed as mean and standard deviation (SD) or median and interquartile range (IQR), based on the distribution of the data. Categorical variables are expressed as counts and percentages.

To compare the differences between subjects before and after the KD intervention, we used the Paired *t*-test for normally distributed data or the Wilcoxon Signed-Rank test for non-normally distributed data, as assessed by the Shapiro–Wilk test. To analyze the effect of diet compliance, Mann–Whitney U tests were used to compare continuous, non-normally distributed outcome variables between the categorical diet compliance groups (e.g., high vs. low compliance). For comparisons of categorical variables between groups, Chi-square tests (or Fisher’s exact test where appropriate) were used.

A *p*-value ≤ 0.05 was considered statistically significant. The magnitude of significant findings was evaluated using effect sizes: Cohen’s d for Paired *t*-tests and Wilcoxon r for Wilcoxon Signed-Rank tests. To assess the precision of our estimates, 95% confidence intervals (CIs) were reported for all primary outcomes. For the Paired *t*-test, we report the 95% CI for the mean difference. For the Wilcoxon Signed-Rank test, we report the 95% CI for the Hodges–Lehmann estimator, which represents the pseudo-median of the paired differences. The robustness of the findings was assessed through sensitivity analyses involving the exclusion of outliers. All statistical analyses were performed using IBM SPSS Statistics v.23 (Armonk, NY, USA).

## 3. Results

Fifty-eight obese children aged 8 to 18 years who followed a “Well-Formulated Ketogenic Diet” were monitored for a period of four months. Before starting and upon completing the diet, multiple anthropometric, biochemical, endocrine, and ultrasound parameters related to obesity, insulin resistance, and metabolic syndrome were assessed. In Table 2, we present the principal anthropometric, biochemical, and laboratory parameters monitored in the entire cohort before the initiation and after the completion of the KD.

### 3.1. Insulin

Baseline evaluation of the patients prior to initiation of the KD demonstrated elevated mean fasting insulin levels (mean value 20.12 μIU/mL). All patients exhibited postprandial hyperinsulinemia during the oral glucose tolerance test (OGTT). Hyperinsulinemia was defined as a more than fivefold increase from baseline insulin levels or values exceeding 100 μIU/mL.

Follow-up assessments after completion of the dietary intervention revealed a statistically significant reduction in fasting insulin levels (*p* < 0.0001) (Table 3). In some of the children with good or moderate compliance to the diet, insulin levels reached normal values (<10 μIU/mL) or values close to the accepted pediatric reference range. Sex-specific analysis demonstrated a significantly greater decrease in baseline insulin levels in girls compared with boys (*p* = 0.02) (Supplementary Data—Table S1). Patients with good or moderate dietary compliance exhibited significantly lower fasting insulin levels following the intervention (Supplementary Data—Table S3).

At the end of the dietary period, OGTT was performed only in one female patient with normal fasting insulinemia in order to assess changes in postprandial insulin secretion; her results are presented in Supplementary Data—Figure S1.

### 3.2. TSH and Thyroid Hormones

TSH, thyroid hormones T3 and T4, as well as antibodies specific to autoimmune Hashimoto’s thyroiditis (anti-thyroid peroxidase antibodies and antithyroglobulin antibodies), were measured in all patients before the start and after the completion of the diet.

Seven of the patients were diagnosed with Hashimoto’s thyroiditis. All of them were euthyroid at baseline (two were receiving L-thyroxine substitution therapy at a dose of 25 μg). Following the KD intervention, a significant reduction in mean serum fT3 levels was observed in the entire cohort (*p* < 0.0001) (Table 3), which was more pronounced among female patients, though independent of age (Supplementary Data—Tables S4 and S5). Concurrently, a slight but significant increase in mean fT4 concentration was recorded (*p* = 0.05) (Table 3), without differences by sex, age groups or dietary compliance (Supplementary Data—Tables S7–S9) No significant changes were found in TSH levels (*p* = 0.13) (Table 3), either in the whole group or when stratified by sex, age, or dietary compliance (Supplementary Data—Tables S10–S12).

At baseline, there were no statistically significant differences in mean TSH, fT3, or fT4 levels between patients with and without autoimmune thyroiditis. By the end of the intervention, however, a significant difference in mean TSH was noted: patients without thyroiditis demonstrated a significant decrease in TSH, whereas those with Hashimoto’s thyroiditis exhibited significantly increased TSH values, with mean levels exceeding the reference range. No significant between-group differences were observed in fT3 or fT4 concentrations (Table 4, Supplementary Data—Tables S13 and S14 and Figure S2).

### 3.3. Patients with PCOS

Eight girls aged 15–17 years met the diagnostic criteria for polycystic ovary syn-drome (according to the Rotterdam consensus) at baseline. All presented with prolonged secondary amenorrhea (>6 months) and required progesterone treatment to induce withdrawal bleeding. None of these patients received hormonal therapy for menstrual regulation during the dietary intervention. All eight girls experienced spontaneous menstrual bleeding within 1–2 months after initiation of the diet, and in some, regular menstrual cycles were established by the end of follow-up. Their individual outcomes are presented in Table 5.

Testosterone was measured in all female patients with clinical signs of advanced pubertal development (n = 17). A statistically significant reduction in mean testosterone levels was observed after completion of the KD intervention (Table 3).

### 3.4. Cortisol

The mean morning cortisol level for the entire cohort also demonstrated a statistically significant decrease at the end of the intervention (*p* = 0.04) (Table 3). Analysis of variance revealed no significant differences when stratified by sex, age or dietary compliance (Supplementary Data—Tables S15–S17).

### 3.5. Adiponectin

Application of the Wilcoxon signed-rank test revealed a significant increase in mean adiponectin levels following the KD intervention (*p* = 0.04) (Table 3). No sex-related differences were observed in mean adiponectin concentrations before and after the intervention (*p* > 0.05) (Supplementary Data—Table S18). However, age-stratified analysis demonstrated significant differences: mean adiponectin levels were highest in the youngest group (8–10 years) and lowest in the oldest group (15–18 years), both at baseline and after completion of the diet (Supplementary Data—Table S19).

Further analysis based on patients’ dietary compliance revealed statistically significant differences in post-intervention adiponectin levels. Specifically, participants demonstrating good or moderate adherence to the ketogenic diet exhibited markedly higher mean adiponectin concentrations at the end of the intervention compared with those with poor compliance, suggesting a dose–response relationship between dietary compliance and the improvement in insulin sensitivity markers (Supplementary Data—Table S20).

## 4. Discussion

In this article, we present the results of the hormonal assessments of the patients from our study, which aimed to investigate the clinical, metabolic, and endocrine effects of a “Well-Formulated Ketogenic Diet” in children with obesity, insulin resistance, and metabolic syndrome. The results regarding the diet’s effect on weight reduction and its impact on symptoms and markers of insulin resistance and metabolic syndrome were presented in our previous article [25]. At the end of the dietary intervention, we observed not only a statistically significant reduction in all anthropometric indicators related to obesity, but also improved insulin sensitivity and a reversal of metabolic syndrome.

Most studies examining the effect of the ketogenic diet in patients with obesity and metabolic disorders demonstrate its effectiveness in reducing body weight, insulin resistance-related markers, and features of metabolic syndrome. Comparatively fewer studies have investigated changes in the levels of other hormones (thyroid hormones, cortisol, adiponectin), which also play a role in the pathogenic processes of obesity. This provided us with the rationale to explore more thoroughly the hormonal changes that occurred as a result of the 4-month ketogenic diet in our patients.

### 4.1. Ketogenic Diet and Insulinemia

Insulin is an anabolic hormone with a central role in metabolic regulation. One of its primary actions is to promote the storage of energy rather than its utilization. The obesogenic effect of insulin has long been recognized, with documented cases of insulin therapy being applied to non-diabetic patients for the purpose of increasing body weight [27]. Even a modest elevation in fasting insulinemia has been shown to markedly suppress lipolysis and stimulate lipogenesis, without significantly inhibiting gluconeogenesis—effects that ultimately favor adipogenesis [27]. Epidemiological studies in children and adolescents link elevated basal insulin (and the concomitant insulin resistance) to greater weight gain later in life [28]. Chronic hyperinsulinemia is not only a determinant in the pathogenesis of obesity but also plays a pivotal role in the development of metabolic syndrome, hepatic steatosis, primary hypertension, and PCOS. Moreover, the associated insulin resistance represents a major contributor to the elevated risk of coronary heart disease and chronic cardiovascular disorders [29,30,31,32,33].

By definition, the KD is characterized by very low intake of digestible carbohydrates, while not requiring excessively high protein consumption. Given that glucose and certain amino acids are the nutrients most potently stimulating insulin secretion [34,35], the reduction in fasting insulinemia observed in our patients was expected. By drastically reducing carbohydrate intake, the KD minimizes postprandial glucose excursions, which directly reduces the stimulus for pancreatic insulin secretion. This carbohydrate restriction shifts hepatic metabolism away from de novo lipogenesis and toward increased fatty acid oxidation and ketogenesis, resulting in decreased hepatic fat accumulation and improved hepatic insulin sensitivity [36,37,38]. Specifically, the ketogenic diet reduces hepatic diacylglycerol content and protein kinase C-ε activity, both of which are implicated in the development of hepatic insulin resistance [36]. A probable role is also played by ketone bodies, such as β-hydroxybutyrate (βHB), produced during ketosis, which have a direct insulin-sensitizing effect in peripheral tissues. In skeletal muscle, βHB alleviates endoplasmic reticulum stress and upregulates the AKT/GSK3β pathway, increasing GLUT4 translocation and glucose uptake, thereby improving insulin sensitivity and reducing circulating insulin requirements [39,40]. The ketogenic diet also suppresses inflammatory signaling and oxidative stress, both of which contribute to insulin resistance. It promotes mitochondrial biogenesis and efficiency, further enhancing insulin sensitivity [36,41,42]. Finally, the ketogenic diet modulates adipose tissue hormones, such as increasing FGF21 and promoting browning of white adipose tissue, which supports improved metabolic stability and insulin sensitivity [42]. It is likely that collectively, these mechanisms result in lower insulin levels by reducing the need for insulin secretion and improving tissue responsiveness to insulin.

In our study, fasting insulin levels were significantly reduced across all age groups, including in the youngest patients (8–10 years), whose baseline insulin levels prior to dietary intervention were within the physiological range. A more pronounced reduction in fasting insulin was observed among children demonstrating better dietary compliance. Notably, the greater compliance recorded among girls likely contributed to the larger decrease in fasting insulinemia within this subgroup.

Repeat OGTT was not incorporated into the follow-up protocol. This test requires several days of prior carbohydrate loading, which was unsuitable for some children who continued the diet for extended periods in order to normalize weight, metabolic, and hormonal imbalances. Consequently, we were unable to systematically evaluate postprandial hyperinsulinemia after the 4-month intervention.

In only one patient, a second OGTT was performed following KD, which revealed a dramatic reduction in postprandial insulin levels (comparative results shown in Supplementary Materials Figure S1). This case concerned an 18-year-old female patient with obesity and polycystic ovary syndrome (PCOS) (patient No. 4 in Table 5). The pronounced reduction in insulin levels following the dietary intervention appears to have been clinically relevant not only for body weight reduction, but also for the observed improvement in menstrual cycle regularity. In this patient, such an association would not have been evident if assessment had been limited to basal insulin levels before and after the intervention. Although this observation is based on a single case and therefore does not permit generalization to the entire study population, it suggests that, in similar clinical settings, the oral glucose tolerance test (OGTT) may provide a more informative assessment of insulinemia and insulin resistance.

Similar findings regarding the impact of KD on insulin levels in obesity have been reported by other authors. Partsalaki et al. compared KD with a low-fat hypocaloric diet in obese children and, after 6 months, demonstrated a significant reduction in fasting insulinemia in both dietary groups [19]. Paoli et al. reported a marked decrease in fasting insulin after 12 weeks of KD in young women with obesity and PCOS [43]. Volek et al. observed reductions in fasting insulin and improved insulin resistance in obese women after only 4 weeks of KD, further proposing that lowered insulin levels may contribute to appetite regulation [17]. The clinical relevance of reduced insulin levels in the context of weight loss and improvement of metabolic disturbances in low-carbohydrate diets has also been emphasized by Staverosky et al., who suggested that diminished insulin secretion itself is a key factor underlying the improvements in markers commonly associated with insulin resistance and metabolic syndrome [18].

In light of these findings, we consider the reduction in basal insulinemia observed in our study to be of paramount importance—not only for weight reduction in our patients, but also as a fundamental mechanism underpinning the observed improvements in insulin sensitivity, normalization of blood pressure in a large proportion of cases, reversal of metabolic syndrome, and the beneficial changes in girls with PCOS.

### 4.2. Ketogenic Diet and Thyroid Function

The ketogenic diet induces profound changes in energy metabolism, shifting from an anabolic insulin-dominant state to a catabolic glucagon-dominant state. This is accompanied by a transition in the primary energy source, with predominant utilization of fats and ketones [21]. These processes are associated with metabolic adaptation and hormonal changes that resemble those observed during fasting [44]. It is well established that fasting affects the hypothalamic–pituitary–thyroid axis, suppressing anabolic activity and reducing the conversion of T4 to T3 [45]. On the other hand, thyroid dysfunction is relatively common among obese patients [46,47,48], raising the important question of how KD influences thyroid hormones. To our knowledge, no studies have examined the effects of KD on thyroid hormones in children with obesity. The few available studies in patients with epilepsy undergoing KD highlight the necessity of monitoring thyroid hormones during such dietary interventions [20,21,49].

In our patients, changes in mean TSH and thyroid hormone levels included a sig-nificant decrease in T3 (*p* < 0.0001), a borderline increase in T4 (*p* = 0.05), and stable TSH levels. These findings are consistent with the observations of other authors investigating the impact of KD on thyroid function.

In a relatively short-term study (12 weeks) of adult patients with epilepsy without prior thyroid disease undergoing a modified Atkins diet, Molteberg et al. reported a significant decrease in T3 and an increase in T4, together with a nonsignificant rise in TSH and rT3. The authors suggested that KD more likely affects peripheral conversion of T4 to T3 rather than central regulatory mechanisms, and recommended monitoring of thyroid hormones during KD treatment [49]. A crossover study in 11 healthy volunteers investigated the effects of two isocaloric diets (KD and a low-fat diet) on thyroid hormones. Each diet was followed for 3 weeks. A significant decrease in T3 and an increase in T4 were observed only after KD, leading the authors to hypothesize that KD induces metabolic changes warranting further investigation [50]. In a 12-month study of children with epilepsy on a strict KD, Yılmaz et al. found no changes in TSH levels at the end of follow-up. At the same time, a significant increase in T4 was observed, including among patients with hypothyroidism receiving replacement therapy prior to KD initiation [21].

In our study, the observed changes in T3 and T4 are likely related to adaptive processes in overall metabolism during KD, substantial weight reduction, and alterations in peripheral conversion of T4 to T3. The hypothesis of altered deiodinase activity requires dedicated investigation, including assessment of rT3, to confirm these mechanisms.

Of note are the findings of Kose et al., who followed 120 children with refractory epilepsy on strict KD for 12 months. Subclinical hypothyroidism was detected in 20 children (16.7%), leading to initiation of replacement therapy. Most of these cases occurred in children with baseline TSH > 5 μIU/mL. The authors identified higher baseline TSH and female sex as independent risk factors for hypothyroidism during KD and recommended thyroid function monitoring [20].

In our cohort, patients without pre-existing thyroid disease remained euthyroid after 4 months of KD. However, in some patients with Hashimoto’s thyroiditis, a significant increase in TSH was detected at the end of the diet, necessitating adjustments in replacement therapy. Since autoimmune thyroiditis is a progressive disease with inevitable development of hypothyroidism, it cannot be conclusively determined that KD was responsible for the deterioration of thyroid function. A limitation of our study is the relatively small sample of patients with confirmed autoimmune thyroid disease in whom elevated TSH was recorded. Thus, coincidental activation of the autoimmune process cannot be excluded. This process is particularly susceptible to fluctuations in the pediatric and adolescent age groups. It is also conceivable that the adaptive metabolic changes induced by KD, requiring adjustments in thyroid hormone secretion and peripheral conversion, may contribute to an inadequate hormonal response in patients with pre-existing thyroid disease.

Our findings, together with those of other authors, support the safety of KD in patients without pre-existing thyroid disease, but they underscore the need for clinical and hormonal monitoring, especially in individuals with established conditions such as hypothyroidism and autoimmune Hashimoto’s thyroiditis.

### 4.3. KD and PCOS

Polycystic ovary syndrome (PCOS) is the most common endocrine disorder among women of reproductive age [51]. In addition to obesity, a large proportion of patients present with symptoms of insulin resistance, metabolic syndrome, or type 2 diabetes mellitus [51,52,53,54]. Epidemiological studies indicate a marked increase in the prevalence of PCOS over the past three decades among adolescents and young women aged 10–24 years [55], a trend that parallels the global obesity epidemic. Due to the limited effectiveness of pharmacological treatments, the identification of suitable and effective lifestyle and nutritional interventions is of particular importance for these patients.

In our study, the eight girls with PCOS demonstrated good to excellent compliance with KD, likely because they perceived it not only as a means of weight loss but also as a therapeutic option for managing their menstrual disturbances. All patients showed significant weight reduction and improved insulin sensitivity, and most exhibited markedly lower testosterone levels. After prolonged amenorrhea and minimal response to hormonal treatment, the appearance of spontaneous menstrual cycles within 1–2 months of initiating KD was a clear therapeutic success.

The etiology of PCOS is not fully understood, but chronic hyperinsulinemia and insulin resistance are considered to play a central role in its pathogenesis. Elevated insulin levels stimulate androgen production in the ovarian theca cells and inhibit hepatic synthesis of sex hormone-binding globulin (SHBG), resulting in increased transport of free androgens to target tissues [56].

The mechanisms by which the KD may improve PCOS are multifactorial and involve several interrelated metabolic and endocrine pathways. Reduced carbohydrate intake, lower circulating insulin levels and improved insulin sensitivity are critical in PCOS, where hyperinsulinemia drives ovarian androgen production and disrupts folliculogenesis. Multiple studies in both humans and animal models demonstrate that KD reduces fasting insulin, HOMA-IR, and improves glycemic control in PCOS [43,57,58,59,60]. By lowering insulin, the KD indirectly decreases ovarian androgen synthesis. Clinical and preclinical data show reductions in total and free testosterone, DHEAS, and the LH/FSH ratio, with concurrent increases in SHBG, further reducing bioavailable androgens [57,61]. The anti-inflammatory effects of KD are well known. The principal ketone body, βHB, exerts anti-inflammatory actions by inhibiting the NLRP3 inflammasome and attenuating NF-κB signaling [62]. This reduces ovarian and systemic inflammation, which is implicated in the pathogenesis of PCOS [62,63]. KD also restores the balance between apoptosis and proliferation in ovarian tissue, reducing follicular atresia and improving ovulatory function, as shown in animal models [63,64]. KD modifies the gut microbiome composition and associated metabolites, which can influence androgen metabolism and systemic inflammation, further contributing to hormonal regulation in PCOS [62,65]. Weight loss and adipose tissue reduction induced by KD are likely to reduce peripheral aromatization of androgens to estrogens and restoration of normal gonadotropin secretion and ovarian function with improvement of the metabolic and reproductive outcomes [43,59].

Collectively, these mechanisms converge to improve metabolic, endocrine, and reproductive parameters in PCOS, with evidence supporting both direct effects of ketosis and indirect effects via weight loss and improved insulin sensitivity.

Following women with PCOS on KD with gradual carbohydrate reintroduction, Rossetti et al. found that the beneficial effects of the diet were independent of weight loss, being also present in normal-weight patients. They suggested that nutritional ketosis itself may contribute to the observed positive outcomes [22].

In our study, the restoration and regulation of menstrual cycles also occurred in most patients before substantial weight loss had been achieved, suggesting that the effect of KD cannot be attributed solely to weight reduction. It is more likely that a combination of factors, such as strict carbohydrate restriction leading to a change in overall metabolism, reduced insulin levels, improved insulin sensitivity, and the anti-inflammatory effect of the ketogenic diet, leads to these positive effects in women with PCOS.

### 4.4. KD and Cortisol

Cortisol is a hormone that plays an important role in obesity, insulin resistance, and metabolic disturbances. Chronically elevated cortisol levels are thought to influence appetite and eating behavior, increase systemic insulin resistance, and thus contribute to many of the abnormalities associated with metabolic syndrome—such as visceral obesity, impaired glucose tolerance, dyslipidemia, and others [66,67,68]. According to some authors, chronic hyperinsulinemia leads to activation of the hypothalamic-pituitary-adrenal (HPA) axis, resulting in “functional hypercortisolism” [66], which, in turn, contributes to the development of visceral obesity and insulin resistance.

Chronic calorie restriction or fasting diets usually result in increased cortisol levels. However, studies by Nakamura et al. suggest that very low-calorie diets do not significantly alter cortisol secretion [69]. Polito et al. examined the effect of a calorie-restricted KD (700–900 kcal/day) in 30 obese men over an 8-week period. The authors reported a significant reduction in morning cortisol levels after the intervention and hypothesized that this may contribute to the beneficial effects of KD in obese patients [70]. In a study involving a small group of obese men, Stimson et al. compared the effects of KD with those of a balanced diet over 4 weeks. Both interventions resulted in significant weight loss, but only KD altered cortisol metabolism through mechanisms independent of weight loss [71].

In our patients, we observed a reduction in morning serum cortisol levels, consistent with the findings reported by other authors noted above, although the diet followed was not strictly very low-calorie. Given the well-established relationship between changes in cortisol levels and alterations in body weight, body composition, and metabolic disturbances, it is likely that the observed cortisol reduction in our cohort is related to the mechanisms that led to improvements in anthropometric and metabolic parameters.

Possible mechanisms by which the ketogenic diet may lower cortisol levels are through modulating the hypothalamic-pituitary-adrenal (HPA) axis, reducing visceral adiposity, and exerting anti-inflammatory and metabolic effects.

First, the ketogenic diet leads to significant reductions in visceral adipose tissue, which is a source of increased local cortisol regeneration via 11β-hydroxysteroid dehy-drogenase type 1 [71]. Decreasing visceral fat reduces this local cortisol production, thereby lowering systemic cortisol levels [70,71]. Additionally, weight loss and improved metabolic parameters associated with the ketogenic diet can attenuate HPA axis activation, as observed by reductions in salivary cortisol after very low-calorie ketogenic diet (VLCKD) interventions in obese individuals [70]. Ketone bodies such as βHB, produced during ketosis, have direct anti-inflammatory effects by inhibiting the NLRP3 inflammasome and histone deacetylases, which may reduce chronic low-grade inflammation and oxidative stress—both of which are known to stimulate the HPA axis and cortisol secretion [72,73]. This anti-inflammatory action may contribute to a lower baseline cortisol output. The KD can indirectly modulate cortisol metabolism through improved insulin sensitivity and glycemic control. Improved insulin sensitivity reduces the compensatory activation of the HPA axis that occurs in states of insulin resistance and metabolic syndrome [74,75]. The KD may influence neuroendocrine signaling, including reductions in sympathetic nervous system activity, which is closely linked to HPA axis tone and cortisol secretion [42]. Evidence indicates that KD reduces the expression of the hypothalamic genes for proopiomelanocortin (POMC), which, together with lower insulin levels, may contribute to decreased cortisol secretion [76].

In summary, the KD may lower cortisol levels via reduction in visceral adiposity, anti-inflammatory effects of ketone bodies, improved insulin sensitivity, and modulation of neuroendocrine pathways, as supported by recent clinical and mechanistic studies [42,70,72,73,74].

### 4.5. KD and Adiponectin

Visceral adipose tissue is particularly active in releasing adipokines and hormones involved in a wide range of metabolic and inflammatory processes. Among them, adiponectin plays a central role, accounting for approximately 0.1% of serum proteins. Since its discovery in 1995, adiponectin has been the focus of numerous studies consistently demonstrating its role in the pathogenesis of obesity, diabetes, systemic inflammation, cardiovascular disease, atherosclerosis, and other conditions [77,78].

The biological functions of adiponectin are diverse. One of its most important physiological effects is the regulation of insulin sensitivity in muscle cells by modulating lipid metabolism (via AMPK, p38MAPK, and PPARα pathways), improving glucose metabolism (through effects on the GLUT4 receptor), and enhancing fatty acid oxidation [79]. Adiponectin inhibits the secretion of leptin and several pro-inflammatory cytokines, such as IL-6 and TNF-α, thereby exerting anti-inflammatory effects [80]. It also improves endothelial cell function by enhancing COX-2 and eNOS activity, leading to increased nitric oxide synthesis [80]. Several studies have found associations between serum adiponectin levels and reproductive health, including disorders such as PCOS, gestational diabetes, pre-eclampsia, and endometriosis [81]. Moreover, adiponectin is known to raise HDL-C levels and lower triglycerides by enhancing the catabolism of triglyceride-rich lipoproteins [82,83,84]. Thus, higher circulating adiponectin levels are considered protective against the development of atherosclerosis [82]. Adiponectin also plays a significant role in appetite regulation and energy balance [78,85].

In a 2005 consensus statement, the International Diabetes Federation (IDF) recommended measuring adiponectin and leptin levels as biomarkers of adipose tissue in order to improve the diagnosis of metabolic syndrome [86].

In our study, a 4-month KD led to a modest but statistically significant increase in mean adiponectin levels (*p* = 0.04), which we consider a marker of improved insulin sensitivity in our patients. This effect of KD was particularly pronounced when outcomes were analyzed by dietary compliance. In the group of children with good adherence to KD, greater reductions in body weight, BMI, and improvements in many clinical and laboratory parameters were accompanied by significantly higher adiponectin levels. No significant differences were observed by sex. The youngest patients showed higher adiponectin levels both before and after the intervention compared to older children, likely reflecting greater insulin sensitivity during prepubertal or early pubertal development.

Although few in number, studies have consistently found that KD leads to increased adiponectin levels—results similar to our observations [19,87,88,89,90,91].

The clinical significance of increased adiponectin levels in the context of improved insulin sensitivity and reversal of metabolic syndrome in our patients raises intriguing questions about the underlying mechanisms by which KD may affect adiponectin secretion.

It is well established that dietary macronutrient composition influences adiponectin levels [92]. A 6-week study in healthy individuals found that a high-carbohydrate, low-fat diet resulted in lower adiponectin levels compared to an isocaloric higher-fat, lower-carbohydrate diet [93]. Other studies demonstrate that the intake of simple sugars, such as glucose and fructose—whose overconsumption is linked to metabolic syndrome—is associated with lower serum adiponectin [94,95]. Diets with severe caloric restriction can also increase adiponectin levels [96], but some authors suggest that this requires energy intake ≤ 50% of daily requirements [97].

Interestingly, KD studies often report increased adiponectin despite not strictly restricting caloric intake, in line with the recommendations of our intervention. Furthermore, patients with epilepsy, who are frequently of normal or even low body weight, still show increased adiponectin levels while maintaining adequate caloric intake. Possible explanations include elevated βHB levels characteristic of KD, which activate the GPR109A receptor, thereby stimulating adiponectin synthesis [98,99]. Another mechanism may involve reduced activation of the NLRP3 inflammasome by circulating βHB, resulting in lower chronic systemic inflammation, which is known to inhibit adiponectin secretion [100]. Other authors highlight the role of free fatty acids, which, via PPARα activation, suppress cytokines such as IL-6 and TNF-α, both inhibitors of adiponectin secretion in adipocytes [101]. Improved mitochondrial function and reduced reactive oxygen species (ROS) production during KD may also play a role [98].

Regardless of the precise mechanisms, we believe that the KD-induced increase in adiponectin levels is likely an important contributor to the improved insulin sensitivity observed in our patients at the end of the dietary intervention.

### 4.6. Limitations and Issues of Concern

We acknowledge that a limitation of our study is the relatively short duration of the dietary intervention, which prevents us from confidently stating whether the hormonal changes we observed would be maintained over a longer period of time.

Another limitation is the relatively small number of children with autoimmune thyroiditis, which means the change in TSH levels may be random rather than diet-induced. The number of girls with PCOS was also relatively small, yet the improvement in their clinical condition and the occurrence of spontaneous menstruation after a long period of secondary amenorrhea in all of them was an undeniable positive effect of the dietary intervention. We also acknowledge that our patients were children of different ages and at different stages of pubertal development, which complicated the interpretation of the results. By analysing each parameter according to sex, age, and dietary compliance, we attempted to determine the impact of these factors on the outcomes of the entire patient cohort.

No significant age- or sex-related differences were observed in thyroid hormones or cortisol levels. This finding is consistent with the fact that, although puberty exerts some influence on these hormones, the changes are generally small and remain within reference ranges. Testosterone was measured and analysed only in girls at advanced stages of pubertal development (i.e., post-menarche). Age-related differences in adiponectin levels are well established, and, as expected, the younger patients exhibited higher concentrations both before and after completion of the dietary intervention. Although some sex-related differences in fasting insulinemia were observed, a significant reduction in basal insulin levels was detected in both girls and boys and across all age groups (the results are shown in Supplementary Materials).

It is also well recognized that hormonal changes in the body occurring in response to atypical metabolic conditions (such as fasting, insulin resistance, etc.) are often adaptive in the short term; however, over prolonged periods, these responses may become maladaptive. Undoubtedly, we believe that clarifying the hormonal effects of KD in obese children requires future studies with larger groups of patients and for longer periods of time.

## 5. Conclusions

A relatively short-term (4-month) “well-formulated ketogenic diet” in children with obesity is associated with hormonal adaptations that promote weight reduction and improve insulin sensitivity, as evidenced by decreased basal insulinemia, lower cortisol levels, and elevated adiponectin concentrations. Notably, the diet demonstrated particularly favorable effects in girls with polycystic ovary syndrome, resulting in the restoration of natural menstrual cycles. These findings suggest that the KD may represent an important component of a comprehensive therapeutic strategy in such cases.

Participants without pre-existing thyroid disorders remained euthyroid at the end of the intervention. The observed alterations in thyroid hormone levels—specifically, a reduction in triiodothyronine (T3) and a slight increase in thyroxine (T4)—are likely attributable to adaptive metabolic responses associated with the predominant utilization of fats as the main energy substrate during ketosis.

Given the high prevalence of Hashimoto’s thyroiditis among individuals with obesity and metabolic syndrome, it is essential to assess thyroid function and autoimmune thyroid markers prior to the initiation of a KD. Moreover, close monitoring of thyroid function and autoimmune activity during dietary intervention is recommended to enable timely adjustment or initiation of hormone replacement therapy when clinically indicated.

## Figures and Tables

**Table 2:** Anthropometric and laboratory parameters of patients who completed the study.

Variable		Before KD (n = 58)	After KD (n = 58)	*p*-Value
Anthropometric indicators	Weight, kg	89.43 ± 3.30	82.98 ± 3.25	<0.0001
	BMI, kg/m^2^	33.35 ± 7.29	30.23 ± 7.14	<0.0001
	Waist circumference, cm	103.97 ± 17.17	91.38 ± 15.03	0.003
	Waist-to-hip ratio	0.64 ± 0.09	0.55 ± 0.08	<0.0001
Laboratory indicators	Fasting glucose (mmol/L)	4.94 ± 0.45	4.80 ± 0.45	0.07
	Fasting insulin (mlU/L)	20.12 ± 8.88	13.17 ± 5.81	<0.0001
	HOMA-IR	4.51 ± 2.20	2.85 ± 1.45	<0.0001
	QUICKI	0.31 ± 0.019	0.33 ± 0.024	<0.0001
	Glycated hemoglobin (HbA1c) %	5.21 ± 0.37	5.30 ± 0.38	0.27
	Total cholesterol (mmol/L)	4.38 ± 0.76	4.26 ± 0.91	0.25
	HDL (mmol/L)	1.17 ± 0.21	1.15 ± 0.22	0.45
	LDL (mmol/L)	2.739 ± 0.68	2.742 ± 0.75	0.97
	Triglycerides (mmol/L)	1.07 ± 0.45	0.88 ± 0.36	0.001
	Triglycerides/HDL	2.22 ± 1.13	1.83 ± 0.87	<0.0001
	Creatinine (mmol/L)	63.48 ± 7.53	63.40 ± 10.19	0.924
	Uric acid (µmol/L)	380.59 ± 90.91	373.36 ± 91.65	0.37
	Urinary calcium/creatinine ratio (mmol/mmol)	0.195 ± 8.88	0.193 ± 5.81	0.71
	ALT (IU/L)	28.87 ± 35.49	22.62 ± 16.21	0.001
	AST (IU/L)	25.82 ± 12.86	23.30 ± 8.54	0.006
	GGT (IU/L)	24.17 ± 12.93	19.62 ± 8.72	<0.0001
	FLI	60.60 ± 29.45	40.67 ± 31.91	<0.0001

Data are presented as mean ± standard deviation (SD). Wilcoxon signed rank test was used to assess change within groups. Mann–Whitney U test was used to compare the change between groups when the data were not normally distributed. *p* < 0.05 was considered significant.

**Table 3:** Mean values of the monitored hormones before and after the KD. Testosterone is measured only in pubertal girls with advanced pubertal development (N = 17).

Hormone	Before the KD	After the KD	Change (95% CI)	*p*-Value	Effect Sizes
Fasting Insulin (mlU/L)	17.70 (13.30–25.93)	11.10 (8.80–15.20)	−6.60 (−9.20; −4.10)	<0.0001 ^b^	0.67
T3 (pg/mL)	6.10 (5.60–6.80)	5.60 (5.10–6.10)	−0.50 (−0.70; −0.30)	<0.0001 ^b^	0.63
T4 (ng/dL)	12.77 ± 1.78	13.20 ± 1.84	+0.34 (+0.01; +0.67)	0.05 ^a^	0.19
TSH (µIU/mL)	3.43 ± 1.58	3.30 ± 2.52	−0.13 (−0.65; +0.38)	0.13 ^a^	-
Cortisol (nmol/L)	396.00 (298.50–480.00)	319.50 (247.75–410.00)	−76.50 (−125.00; −30.00)	0.04 ^b^	0.48
Adiponectin (mcg/mL)	8.20 (6.30–10.20)	8.70 (6.80–10.80)	+0.50 (+0.10; +1.20)	0.04 ^b^	0.40
Testosterone (ng/mL)	0.74 (0.54–0.93)	0.64 (0.48–0.81)	−0.10 (−0.15; −0.05)	0.01 ^b^	0.57

Data are presented as mean ± standard deviation (SD) for normally distributed (T4, TSH) and median and interquartile range (IQR) for non-normally distributed (Fasting Insulin, T3, Cortisol, Adiponectin, Testosterone). *p*-values were derived using ^a^ Paired Samples *t*-test for normally distributed data, ^b^ Wilcoxon Signed-Rank test for non-normally distributed data. *p* ≤ 0.05 was considered significant. The Change (95% CI) reports the mean difference for Paired Samples *t*-test or the Hodges–Lehmann median difference for Wilcoxon Signed-Rank test and its 95% confidence interval (CI) to indicate the precision of the estimate. Effect sizes were calculated to quantify the magnitude of significant changes. For the Wilcoxon Signed-Rank Test, the effect size is reported as Wilcoxon r and Cohen’s d for the Paired *t*-test. Effect sizes are interpreted as: r/d = 0.1 (small), 0.3 (medium), 0.5 (large). Effect size is not typically reported for non-significant results.

**Table 4:** Thyroid hormones before starting and after completing the diet in patients with and without Hashimoto’s autoimmune thyroiditis.

Hormone	Group of Patients	N	Mean ± SD	95% CI	*p*-Value	Effect Sizes
TSH before the KD (µIU/mL)	Patients without Hashimoto’s Thyroiditis	51	3.36 ± 1.60	(−1.27; 0.09)	*p* > 0.05 ^a^	-
	Patients with Hashimoto’s Thyroiditis	7	3.95 ± 1.42			
TSH after the KD (µIU/mL)	Patients without Hashimoto’s Thyroiditis	51	2.89 ± 1.50	(−5.67; −1.11)	*p* = 0.001 ^a^	0.94
	Patients with Hashimoto’s Thyroiditis	7	6.28 ± 5.47			
T3 before the KD (pg/mL)	Patients without Hashimoto’s Thyroiditis	51	6.26 ± 1.05	(−0.55; 0.73)	*p* > 0.05 ^b^	-
	Patients with Hashimoto’s Thyroiditis	7	6.17 ± 0.97			
T3 after the KD (pg/mL)	Patients without Hashimoto’s Thyroiditis	51	5.65 ± 0.89	(−0.28; 0.62)	*p* > 0.05 ^b^	-
	Patients with Hashimoto’s Thyroiditis	7	5.48 ± 0.68			
T4 before the KD (ng/dL)	Patients without Hashimoto’s Thyroiditis	51	12.83 ± 1.81	(−0.73; 1.71)	*p* > 0.05 ^a^	-
	Patients with Hashimoto’s Thyroiditis	7	12.34 ± 1.60			
T4 after the KD (ng/dL)	Patients without Hashimoto’s Thyroiditis	51	13.32 ± 1.91	(0.18; 1.88)	*p* > 0.05 ^a^	-
	Patients with Hashimoto’s Thyroiditis	7	12.29 ± 0.93			

Data are presented as mean ± standard deviation (SD). *p*-values were derived using ^a^ Paired Samples *t*-test for normally distributed data, ^b^ Wilcoxon Signed-Rank test for non-normally distributed data. *p* ≤ 0.05 was considered significant. Effect sizes were calculated to quantify the magnitude of significant changes. For the Wilcoxon Signed-Rank Test, the effect size is reported as Wilcoxon r and Cohen’s d for the Paired *t*-test. Effect size is not typically reported for non-significant results.

**Table 5:** Clinical and biochemical parameters before and after KD in female patients with PCOS (N = 8). The ketogenic diet (KD) led to rapid resumption of menstruation, weight loss, and marked reductions in insulin and testosterone, with the greatest improvements seen in patients with the highest baseline metabolic and hormonal disturbances, highlighting the KD’s effectiveness in addressing hyperinsulinemia and hyperandrogenism.

Parameter	N1	N2	N3	N4	N5	N6	N7	N8
Period of amenorrhoea before KD	6 months	6 months	>1 year	1.5 years	7 months	1 year	>1 year	6 months
Spontaneous menstruation after KD	After 1 month	After 1 month	After 1 month	After 2 months	After 2 months	After 1 month	After 2 months	After 2 months
Testosterone before KD (ng/mL)	0.73	0.67	2.20	0.69	0.74	1.07	0.86	0.70
Testosterone after KD (ng/mL)	0.57	0.24	1.73	0.49	0.33	0.96	0.85	0.71
Fasting insulin before KD (μIU/mL)	25.14	10.52	49.96	10.42	15.23	41.19	44.57	11.26
Fasting insulin after KD (μIU/mL)	23.40	7.15	8.43	6.10	12.82	16.27	26.70	5.01
Weight loss (%)	−13.7%	−12.8%	−12.4%	−8.5%	−9.6%	−7.8%	−5.8%	−4.7%
Menstrual cycle after KD	regular	regular	regular	irregular	irregular	regular	irregular	irregular

## Data Availability

Data will be made available upon reasonable request to the corresponding author due to privacy restrictions.

## Supplementary Materials

The following supporting information can be downloaded at: https://www.mdpi.com/article/10.3390/children13030406/s1, Table S1: Fasting insulin before and after the KD by gender; Table S2: Fasting insulin before and after the KD by age; Table S3: Fasting insulin after the KD according to compliance with the diet; Table S4: T3 before and after the KD by gender; Table S5: T3 before and after the KD by age; Table S6: T3 after the KD according to the compliance with the diet; Table S7: T4 before and after the KD by gender; Table S8: T4 before and after the KD by age; Table S9: T4 after the diet according to the compliance with the diet; Table S10: TSH before and after the KD by age; Table S11: TSH before and after the KD by age; Table S12: TSH after the diet according to the compliance with the diet; Table S13: TSH and thyroid hormones in patients with and without Hashimoto’s thyroiditis before the KD; Table S14: TSH and thyroid hormones in patients with and without Hashimoto’s thy-roiditis after the KD; Table S15: Cortisol before and after the KD by gender; Table S16: Cortisol before and after the KD by age; Table S17: Cortisol after the diet according to the compliance with the diet; Table S18: Adiponectin before and after the KD by gender; Table S19: Adiponectin before and after the KD by age; Table S20: Adiponectin after the KD according to the compliance with the diet; Figure S1: Changes in insulin levels during the OGTT in an 18-year-old female patient with PCOS; Figure S2: Changes in TSH, T3 and T4 mean concentrations before and after the KD in patients with and without Hashimoto’s thyroiditis.

## Abbreviations

The following abbreviations are used in this manuscript:

KD	Ketogenic diet
PCOS	Polycystic ovary syndrome
OGTT	Oral glucose tolerance test
βHB	β-hydroxybutyrate
HPA axis	Hypothalamic-pituitary-adrenal axis
SHBG	Sex hormone-binding globulin
