# Supplementary material for: Low-Carbohydrate (Ketogenic) Diet in Children with Obesity: Part 2—Hormonal Effects of the Ketogenic Diet
Source: Children (Basel). 2026 Mar 14;13(3):406. doi: 10.3390/children13030406 (PMC13024901; doi:10.3390/children13030406)

Supplementary Materials

1. Fasting insulin

Table S1. Fasting insulin before and after the KD by gender

Fasting insulin in mlU/l / gender	N	Gender	Mean	SD	*F, df, P*
Fasting insulin in mlU/l before the diet	35	boys	20.45	±7.17	F=0.11 df=1 p=0.73
	23	girls	19.62	±11.16	
Fasting insulin in mlU/l after the diet	35	boys	14.57	±5.72	F=5.49 df=1 p=0.02
	23	girls	11.05	±5.41	

Table S2. Fasting insulin before and after the KD by age

Fasting insulin in mlU/l / age group	N	Age group	Mean	SD	*F, df, P*
Fasting insulin in mlU/l before the diet	8	8-10 years	14.39	±3.25	F=2.10
	25	11-15 years	20.50	±7.69	df=2
	25	16-18 years	21.58	±10.57	p=0.13
Fasting insulin in mlU/l after the diet	8	8-10 years	9.17	±1.96	F=2.51
	25	11-15 years	14.32	±6.61	df=2
	25	16-18 years	13.31	±5.37	p=0.09

Table S3. Fasting insulin after the KD according to compliance with the diet

Fasting insulin in mlU/l after the diet	N	Mean	SD	95% CI for Mean	
				Lower Bound	Upper Bound
Good compliance	26	11.45	±5.41	9.26	13.64
Moderate compliance	15	12.13	±4.48	9.64	14.61
Poor compliance	17	16.73	±6.16	13.56	19.90

2. TSH and thyroid hormones

- T3 (pg/mL)

Table S4. T3 before and after the KD by gender

T3/ gender	N	Gender	Mean	SD	*F, df, P*
T3 before the diet	35	boys	6.43	±0.98	F=2.55 df=1 p=0.12
	23	girls	5.99	±1.07	
T3 after the diet	35	boys	5.99	±0.80	F=20.41 df=1 P<0.0001
	23	girls	5.09	±0.65	

Table S5. T3 before and after the KD by age

T3 / age group	N	Age group	Mean	SD	*F, df, P*
T3 before the diet	8	8-10 years	7.19	±0.92	F=5.17
	25	11-15 years	6.27	±1.05	df=2
	25	16-18 years	5.93	±0.88	p=0.009
T3 after the diet	8	8-10 years	6.13	±0.82	F=2.77
	25	11-15 years	5.73	±0.80	df=2
	25	16-18 years	5.38	±0.88	p=0.07

Table S6. T3 after the KD according to the compliance with the diet

T3 after the diet / compliance with the diet	N	Mean	SD	95% % CI for Mean	
				Lower Bound	Upper Bound
Good compliance	26	5.38	±0.87	5.03	5.73
Moderate compliance	15	5.79	±0.91	5.28	6.29
Poor compliance	17	5.88	±0.74	5.50	6.26

- T4 (ng/dL)

Table S7. T4 before and after the KD by gender

T4/ gender	N	Gender	Mean	SD	*F, df, P*
T4 before the diet	35	boys	12.99	±1.77	F=1.38<br>df=1<br>p=0.25
	23	girls	12.43	±1.80	
T4 after the diet	35	boys	12.90	±1.86	F=2.40<br>df=1<br>P=0.13
	23	girls	13.66	±1.75	

Table S8. T4 before and after the KD by age

T4 / age group	N	Age group	Mean	SD	*F, df, P*
T4 before the diet	8	8-10 years	12.74	±1.67	F=0.01
	25	11-15 years	12.74	±1.61	df=2
	25	16-18 years	12.81	±2.04	p=0.99
T4 after the diet	8	8-10 years	13.72	±2.31	F=1.71
	25	11-15 years	12.69	±1.80	df=2
	25	16-18 years	13.54	±1.66	p=0.18

Table S9. T4 after the diet according to the compliance with the diet

T3 after the KD / compliance with the diet	N	Mean	SD	95% CI for Mean	
				Lower Bound	Upper Bound
Good compliance	26	13.43	±2.01	12.62	14.25
Moderate compliance	15	13.05	±1.11	12.43	13.66
Poor compliance	17	12.97	±2.13	11.88	14.07

- TSH (µIU/mL)

Table S10. TSH before and after the KD by age

TSH/ gender	N	Gender	Mean	SD	*F, df, P*
TSH before the diet	35	boys	3.84	±1.76	F=6.27<br>df=1<br>p=0.01
	23	girls	2.82	±1.00	
TSH after the diet	35	boys	3.60	±2.29	F=1.24<br>df=1<br>P=0.27
	23	girls	2.84	±2.84	

Table S11. TSH before and after the KD by age

TSH / age group	N	Age group	Mean	SD	*F, df, P*
TSH before the diet	8	8-10 years	3.09	±1.21	F=2.78<br>df=2<br>p=0.07
	25	11-15 years	3.98	±1.99	
	25	16-18 years	3.00	±0.98	
TSH after the diet	8	8-10 years	3.19	±1.88	F=0.12<br>df=2<br>p=0.88
	25	11-15 years	3.14	±2.33	
	25	16-18 years	3.49	±2.93	

Table S12. TSH after the diet according to the compliance with the diet

TSH after the diet / compliance with the diet	N	Mean	SD	95% CI for Mean	
				Lower Bound	Upper Bound
Good compliance	26	3.74	±1.90	2.30	4.94
Moderate compliance	15	2.99	±1.28	1.92	4.19
Poor comliance	17	3.36	±1.20	2.29	3.75

Table S13. TSH and thyroid hormones in patients with and without Hashimoto's thyroiditis before the KD

Hormones	Groups of patients	Mean	SD	N
TSH before the diet	Patients without Hashimoto's thyroiditis	3.36	1.60	51
	Patients with Hashimoto's thyroiditis	3.95	1.42	7
	Total	3.43	1.58	58
T3 before the diet	Patients without Hashimoto's thyroiditis	6.26	1.05	51
	Patients with Hashimoto's thyroiditis	6.17	.97	7
	Total	6.25	1.03	58
T4 before the diet	Patients without Hashimoto's thyroiditis	12.83	1.81	51
	Patients with Hashimoto's thyroiditis	12.34	1.60	7
	Total	12.77	1.78	58

Table S14. TSH and thyroid hormones in patients with and without Hashimoto's thyroiditis after the KD

Hormones	*Groups of patients*	*N*	*Mean*	*SD*	*F, df, P*
TSH after the diet	Patients without Hashimoto's thyroiditis	51	2.89	±1.50	F=13.58 df=1 p=0.001
	Patients with Hashimoto's thyroiditis	7	6.28	±5.47	
T3 after the diet	Patients without Hashimoto's thyroiditis	51	5.65	±0.89	F=0.24 df=1 p=0.63
	Patients with Hashimoto's thyroiditis	7	5.48	±0.68	
T4 after the diet	Patients without Hashimoto's thyroiditis	51	13.32	±1.91	F=1.95 df=1 p=0.17
	Patients with Hashimoto's thyroiditis	7	12.29	±0.93	

3. Cortisol (nmol/l)

Table S15. Cortisol before and after the KD by gender

Cortisol / gender	N	Gender	Mean	SD	*F, df, P*
Cortisol before the diet	35	boys	418.00	±140.03	F=1.62 df=1 p=0.21
	23	girls	368.55	±152.07	
Cortisol after the diet	35	boys	364.55	±193.16	F=0.82 df=1 p=0.37
	23	girls	318.88	±178.12	

Table S16. Cortisol before and after the KD by age

Cortisol / age group	N	Age group	Mean	SD	*F, df, P*
Cortisol before the diet	8	8-10 years	350.59	±205.29	F=1.33
	25	11-15 years	379.71	±127.48	df=2
	25	16-18 years	432.37	±139.71	p=0.27
Cortisol after the diet	8	8-10 years	333.62	±226.20	F=0.49
	25	11-15 years	322.63	±172.45	df=2
	25	16-18 years	374.35	±192.42	p=0.61

Table S17. Cortisol after the diet according to the compliance with the diet

Cortisol after the diet / compliance with the diet	N	Mean	SD	95% CI for Mean	
				Lower Bound	Upper Bound
Good compliance	26	369.64	±200.70	288.58	450.71
Moderate comliance	15	287.66	±190.80	182.00	393.32
Poor compliance	17	362.82	±159.78	280.67	444.97

4. Adiponectin

Table S18. Adiponectin before and after the KD by gender

Adiponectin/ gender	N	Gender	Mean	SD	*F, df, P*
Adiponectin before the diet	35	boys	8.70	±3.30	F=0.05<br>df=1<br>p=0.81
	23	girls	8.47	±4.11	
Adiponectin after the diet	35	boys	9.07	±3.99	F=0.02<br>df=1<br>p=0.87
	23	girls	9.23	±3.74	

Table S19. Adiponectin before and after the KD by age

Adiponectin/ age group	N	Age group	Mean	SD	*F, df, P*
Adiponectin before the diet	8	8-10 years	11.66	±3.55	F=7.16
	25	11-15 years	9.28	±3.30	df=2
	25	16-18 years	6.96	±3.17	p=0.002
Adiponectin after the diet	8	8-10 years	11.64	±3.55	F=5.25
	25	11-15 years	10.04	±3.79	df=2
	25	16-18 years	7.44	±3.37	p=0.007

Table S20. Adiponectin after the KD according to the compliance with the diet

Adiponectin after the diet	N	Mean	SD	95% CI for Mean	
				Lower Bound	Upper Bound
Good compliance	26	10.33	±3.47	8.93	11.74
Moderate compliance	15	9.89	±3.53	7.93	11.84
Poor compliance	17	6.65	±3.72	4.74	8.57

Figure S1. Changes in insulin levels during the OGTT in an 18-year-old female patient with PCOS.

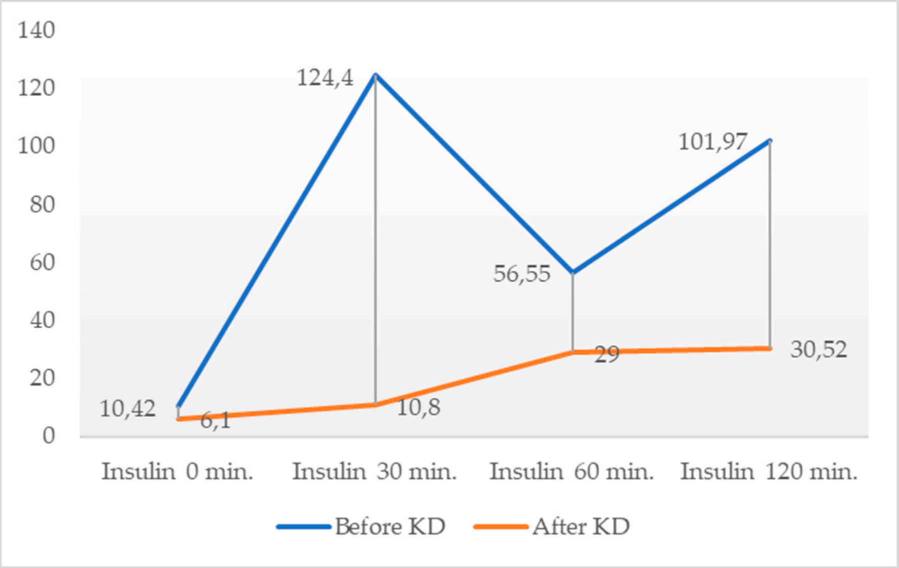

Figure S2. Changes in TSH, T3 and T4 mean concentrations before and after the KD in patients with and without Hashimoto's thyroiditis. (a) Thyroid-stimulating hormone (TSH), (b) triiodothyronine (T3), and (c) thyroxine (T4) levels measured at baseline and at the end of the dietary intervention. Blue lines represent patients with Hashimoto's thyroiditis, and red lines represent patients without Hashimoto's thyroiditis. Each line illustrates the mean change within each group across the two time points. These data demonstrate a divergent response in TSH between groups, with comparatively stable or modest changes observed in T3 and T4 concentrations.

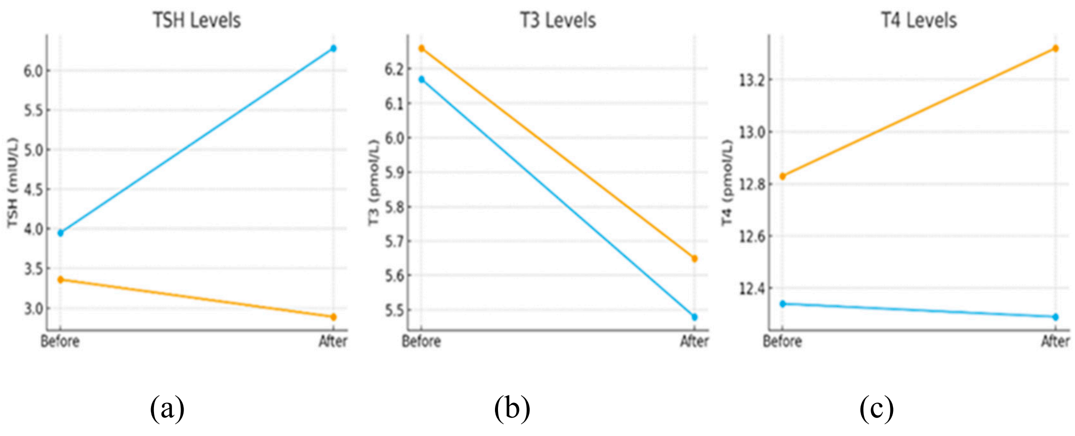

Supplement: Supplementary file 1 [file children-13-00406-s001.zip › children-4164217-supplementary.pdf]
